# AmBisome^®^ Formulations for Pediatrics: Stability, Cytotoxicity, and Cost-Effectiveness Studies

**DOI:** 10.3390/pharmaceutics16040466

**Published:** 2024-03-27

**Authors:** Guendalina Zuccari, Carla Villa, Valentina Iurilli, Paola Barabino, Alessia Zorzoli, Danilo Marimpietri, Debora Caviglia, Eleonora Russo

**Affiliations:** 1 Department of Pharmacy, University of Genoa, Viale Benedetto XV, 16132 Genoa, Italy; carla.villa@unige.it (C.V.); debora.caviglia@edu.unige.it (D.C.); 2 UOC—Unità Operativa Complessa, IRCCS Istituto Giannina Gaslini, via Gerolamo Gaslini 5, 16147 Genoa, Italy; valentinaiurilli@gaslini.org (V.I.); paolabarabino@gaslini.org (P.B.); 3 Stem Cell Laboratory and Cell Therapy Center, IRCCS Istituto Giannina Gaslini, via Gerolamo Gaslini 5, 16147 Genoa, Italy; alessiazorzoli@gaslini.org (A.Z.); danilomarimpietri@gaslini.org (D.M.)

**Keywords:** AmBisome^®^, liposomes, amphotericin B, hospital pharmacy, compounding, pediatric formulations, medicines for children, heath expenditure

## Abstract

Liposomal amphotericin B (Ambisome^®^) is the gold standard for the treatment and prevention of fungal infections both in the adult and pediatric populations. The lyophilized dosage form has to be reconstituted and diluted by hospital staff, but its management can be challenging due to the spontaneous tendency of amphotericin B to form aggregates with different biological activity. In this study, the colloidal stability of the liposomes and the chemical stability of amphotericin B were investigated over time at storage conditions. Three liposomal formulations of amphotericin B at 4.0 mg/mL, 2.0 mg/mL, and 0.2 mg/mL were prepared and assayed for changes regarding the dimensional distribution, zeta potential, drug aggregation state, and onset of by-products. Our analyses highlighted that the most diluted formulation, kept at room temperature, showed the greatest changes in the aggregation state of the drug and accordingly the highest cytotoxicity. These findings are clinically relevant since the lower dosages are addressed to the more vulnerable patients. Therefore, the centralization of the dilution of AmBisome^®^ at the pharmacy is of fundamental importance for assuring patient safety, and at the same time for reducing medication waste, as we demonstrated using the cost-saving analysis of drug expense per therapy carried out at the G. Gaslini children hospital.

## 1. Introduction

**Figure 1:**
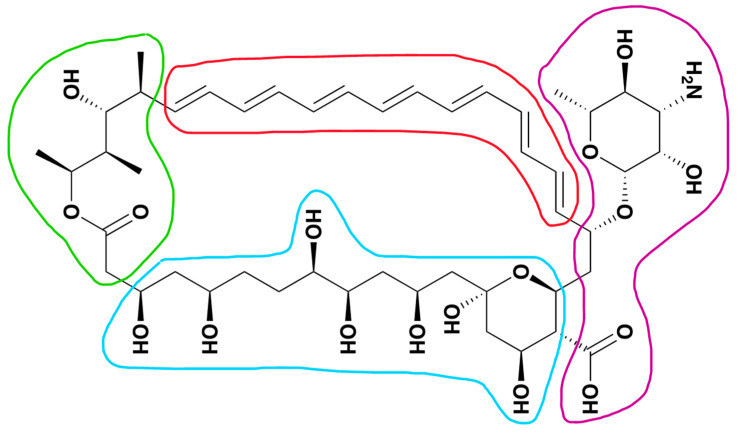
Chemical structure of AmB: polyene chain (red loop), polyol chain (light blue loop), polar tail (green loop), and zwitterionic head (purple loop).

Amphotericin B (AmB) is a macrolide polyene obtained from *Streptomyces nodosus* effective against most clinically relevant fungal species. It is one of the oldest antifungal drugs; nevertheless, up to now it remains still the gold standard of antifungal therapy thanks to its broad-spectrum activity and absence of induction of resistance [1]. Its mechanisms of action have been extensively studied, and it is acknowledged that AmB forms pores in cell membranes leading to an electrolytic imbalance and loss of essential cellular components. This efficiency in permeabilizing cell membranes is associated with a greater affinity of the drug towards ergosterol than cholesterol, leading to a high cytotoxicity towards pathogens. However, to date, all the mechanisms involved are not fully understood, and several hypotheses have been put forward [2]. Indeed, AmB can enter the cells and trigger overproduction of reactive oxygen species (ROS); thus, the increased level of these free-radicals and the imbalance of ions produce multiple detrimental effects on the cellular components (membrane, mitochondria, proteins, and DNA), resulting in fungal cell death [3]. Unfortunately, severe toxic effects are associated with AmB administration, particularly towards red blood cells and kidney cells, and the balance between toxicity/activity seems to be ascribable to the spontaneous aggregation of the drug. It has been reported that the activity of the drug is tuned by the different states of aggregation of the molecule, which spontaneously self-assembles, forming aggregates that are more water soluble [4]. As depicted in Figure 1, the chemical structure of AmB can be divided into four parts: polyene chain, polyol chain, polar tail, and polar hydrophilic zwitterionic head encompassing the mycosamine moiety and the carboxyl group.

Notably, at submicromolar concentrations, the monomers associate in oligomers, usually dimers, even though it is believed that they can contain between four and eight molecules of the drug [4]. Particularly, the drug’s tendency for self-aggregation into dimers is driven by hydrophobic forces that lead to the formation of parallel or antiparallel dimers, while the dipole–dipole interaction between zwitterionic head groups is mostly irrelevant for the association of AmB monomers, even though it possibly modulates the parallel–antiparallel equilibrium [4]. Moreover, Zielińska et al., on the basis of previous studies [5,6], suggested that AmB monomers are capable of permeabilizing only fungal membranes, while AmB dimers are responsible for the side effects due to the interactions with cholesterol-rich membranes. Indeed, the double bond present in the lateral chain of ergosterol represents the structural element responsible for its higher affinity for AmB and consequently for its selective toxicity against fungi [6]. Particularly from studies on fungal membrane, it was found that AmB firstly binds to cell membranes and then forms complexes with ergosterol [7]. More recently, it was demonstrated that AmB−ergosterol complex spans a lipid bilayer with single-molecule length [8]. This result suggests that AmB’s antifungal activity lies in its ability to form membrane pores due to aggregation of AmB−ergosterol complexes. Moreover, the effect of the aggregation state of AmB on its selectivity of interaction with ergosterol with respect to cholesterol was also demonstrated. The monomeric form (i.e., at concentrations < 10^−7^ M, below the CMC) of AmB interacts selectively with ergosterol-containing and not with cholesterol-containing monolayers. On the contrary, above CMC, AmB can promote the extraction of cholesterol from the membrane and, thus, cause toxic effects. Rivnay et al. speculated that the monomeric form of AmB being present in negligible concentrations, as the drug is poorly soluble in both aqueous and highly hydrophobic media (membrane bilayers), its contribution to drug toxicity could be quite irrelevant [9].

Conversely, an increase in solubility associated with a reduction of toxicity was achieved with the transition from homo-aggregates (i.e., oligomers of pure AmB) to hetero-aggregates containing surfactants or lipids [10]. Such structures were found to be less likely to form pores in cholesterol-containing membranes, thus reducing the onset of adverse effects typically presented by the free drug. The first nano-formulation, marketed in 1958, was a combination of AmB with deoxycholate (Fungizone^®^); subsequently, in the 1990s, further efforts were made to ameliorate the therapeutic efficacy and lipid complex with two phospholipids (Abelcet^®^), liposomal AmB (AmBisome^®^), and colloidal dispersion with cholesteryl sodium sulphate (Amphotec^®^), which reached the market [10]. Simultaneously, researchers had been developing several nano-based drug-delivery systems such as polymeric nanoparticles, conjugates, lipid-based delivery systems, nano-emulsions, and nano-suspensions [11].

To date, among all the injectable formulations studied and proposed, AmBisome^®^ is considered the gold standard for the treatment of visceral leishmaniasis and it is indicated for prophylaxis for invasive fungal infection in patients receiving immunosuppressive therapy, for empiric therapy in prolonged febrile neutropenia, invasive aspergillosis and candidiasis, and for the treatment of Cryptococcal meningitis in HIV-infected patients, both for the adult and pediatric populations [12]. In 2021, global sales of AmBisome^®^ reached USD 540 million and are expected to amplify [13]. Although the increasing incidence of life-threatening infections and hospital-acquired infections are the major growth driver for the AmBisome^®^’s sales, the rising demand for targeted therapies together with the increasing number of approved indications are adjunctive factors that contribute to its high market success.

However, it should be stressed that liposomal AmB is difficult and expensive to manufacture and most of the global supply is produced by a single company. To drive down the price, bioequivalent formulations are under development, but, to date, no EMA (European Medicines Agency)-approved generic version is available on the market. As stated by the board of the European Confederation of Medical Mycology (ECMM), there is an unmet need in Europe and beyond for more affordable lipid-based AmB nano-formulations [14]. To support this requirement, EMA developed a product-specific document on the demonstration of equivalent efficacy and safety of a liposomal AmB formulation: “Liposomal amphotericin B powder for dispersion for infusion 50 mg product-specific bioequivalence guidance” [15]. Scientific experts have been questioning for a long time how to evaluate generic liposomal formulations. In this regard, in 2013, EMA released a reflection paper to support a marketing authorization of intravenous liposomal generic product [16]. In this context, several research works have been published to validate the comparability of liposomal formulations. Indeed, it has been already demonstrated that even if the qualitative and quantitative liposomal compositions are identical, the toxicity of the formulation could be higher than that of the originator if the manufacturing process is different, as seen for Anfogen^®^ and Lambin^®^. The hypothetical reason for this difference probably lies in the existence of a significant difference in the drug aggregation state [9].

In recent years, despite the AmB therapeutic index having been improved by its encapsulation in the liposomal delivery system, concerns associated with the administration of this medicine still persist. The handling and managing of AmBisome^®^ by health care staff can be challenging. One case has been reported of confusion between AmBisome^®^ and Fungizone^®^ which led to the rehospitalization of a 9-year-old patient [17], and the utilization of an order panel was encouraged for safe ordering and administration of AmB [18]. Although most are familiar with the high-alert and look-alike/sound-alike concerns, this medication poses other significant risks as well. Anaphylactic-like and infusion reactions have also been reported with all formulations of AmB [18]. In addition, AmB is incompatible with sodium chloride; therefore, it can be challenging to recall the appropriate sequencing of the hydration therapy. Another relevant issue is the pharmacoeconomic aspect related to the rational and cost-effective use of the liposomal formulation. On the basis of these considerations, from May 2022, the hospital pharmacy of the G. Gaslini Children Hospital (Genoa, Italy) considered restricting the preparation of AmBisome^®^ to the pharmacy (i.e., vials unavailable in floor stock) with the aim to better preserve a check system, and to optimize dispensing and scheduling. It is well understood that the colloidal stability of nanoparticles is an important parameter that can directly affect the safety and the efficacy of the drug. For all these reasons, we evaluated the colloidal stability of AmBisome^®^ under in-use storage times undertaken in the hospital pharmacy, thus supporting the attempt of the pharmacists to warrant clinical benefits to patients in a cost-effective manner. The aim of this research was to investigate the colloidal stability and the cytotoxic activity of AmBisome^®^ formulations both in sterile water or in 5% glucose solution maintained at room temperature or at 4 °C up to one week from reconstitution, for better guiding the clinical use of AmBisome^®^. Chemical stability was also monitored by HPLC-DAD analysis to evaluate the possibility of extending the expiration date for use of AmB. Finally, we also analyzed the number of vials dispensed before and after the decision of using the pharmacist-provided medication therapy-management approach in order to establish if the procedure led to a minimization of the costs.

## 2. Materials and Methods

### 2.1. Materials

AmBisome^®^ was purchased from Gilead Sciences Srl (Milan, Italy), sterile water was purchased from Eurospital S.p.A. (Trieste, Italy), and 5% glucose solution for infusion was from Fresenius Kabi (Isola della Scala, Verona, Italy). All solvents and reagents were of HPLC grade and purchased from Merck KGaA (Darmstadt, Germany).

### 2.2. Reconstitution and Dilution of Ambisome^®^ in the Hospital Pharmacy

**Figure 2:**
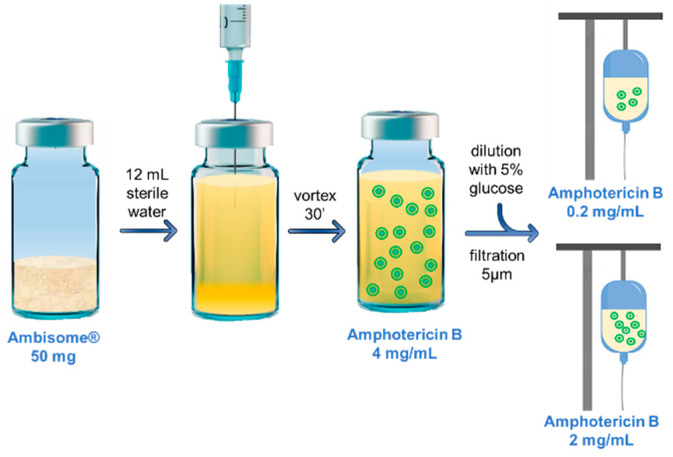
Sketch of reconstitution and dilution of the liposomal suspension to be infused in patients.

The reconstitution instructions and the procedures for dose adjustment are disclosed in the Summary of Product Characteristics (SmPC) agreed with Italian Medicines Agency (AIFA) [19]. Briefly, the lyophilized yellowish powder was reconstituted using 12 mL sterile water so as to have a drug concentration of 4 mg/mL AmB. Then, the liposomal suspension was vortexed for 30 s until aggregates dissolved and homogeneity was reached. The infusion to be administered in patients was obtained by transferring an aliquot of the suspension with a sterile syringe equipped with a 5 µm filter into an infusion bag containing 5% glucose whose volume had been previously adjusted so as to have a final concentration of drug ranging from 0.2 to 2 mg/mL, according to patient’s weight (Figure 2).

### 2.3. Sample Preparation

According to manufacturer’s instructions, AmBisome^®^ is a single-dose unpreserved sterile lyophilized powder that can be stored and administered without protection from light. Once reconstituted, the drug must be used immediately to avoid microbiological contaminations; modifications of in-use storage times and conditions are under the responsibility of the users. However, when the handling of the product is conducted under validated aseptic conditions, the manufacturer gives some indications. In Table 1, the in-use storage times after opening AmBisome^®^ suggested by the manufacturer and those employed in the hospital pharmacy are summarized.

### 2.4. UV–Visible Spectrophotometry for Qualitative Analysis

The aggregation states of AmB can be identified using an UV–visible spectrophotometer (Evolution 300, ThermoFisher Scientific, Segrate, Italy), with a quartz cuvette with a path length of 1 cm. In order to determine the predominant aggregation state of Amb formulations under storage conditions, AmBisome^®^ was reconstituted in sterile water and diluted as described above to obtain three formulations containing 4, 2, or 0.2 mg/mL AmB each. The resulting suspensions were scanned between 300 and 450 nm wavelengths at different time points (0, 1, 2, 3, and 7 days). The analysis was performed both on samples maintained at room temperature or at 4 °C. Three parameters were extracted for the characterization of the formulations: λ_max_ (main peak position, 322 nm); main peak width (at 2/3 height) reflecting heterogeneity of the species; and peak ratio. The ratio was calculated by dividing the absorbance of the first peak (322 nm), characteristic of the oligomer, by the absorbance of the fourth peak (416 nm), representative of the monomer [20]. The three liposomal formulations obtained after reconstitution and dilution with sterile water and 5% glucose (according to manufacturer’s instruction) were further diluted with deionized water, prior spectrophotometric analysis, to 8.7–22.0 µM to assess the oligomer/monomer ratio. Data represent the mean ± SD of three independent experiments.

### 2.5. In Vitro Colloidal Stability of Liposomal AmB Formulations

Mean diameter (Z-average), polydispersity index (PDI), and zeta potential (ƺ) of the liposomal formulations were recorded at 25 °C using a Malvern Nano ZS90 light scattering apparatus (Malvern Instruments Ltd., Worcestershire, UK) at a scattering angle of 90°. The apparent equivalent hydrodynamic radii of the liposomes were calculated using the Stokes–Einstein equation. For the measurements, aliquots of liposomal formulations were withdrawn and diluted when necessary to 0.2 mg/mL AmB with sterile water or 5% glucose solution according to the type of medium present in the sample. The results from these light scattering experiments were presented as the average values ± SD obtained from three different batches and carrying out three runs of ten measurements per sample. The in vitro colloidal stability of reconstituted formulations was also studied by Nanoparticle Tracking Analysis (NTA) using a Nanosight NS300 (Malvern) apparatus equipped with a 352 nm green laser, according to the manufacturer’s software manual [21]. Briefly, samples were diluted in PBS to reach the ideal measurement concentration within a range of 20–100 particles per frame by preliminary tests. For this purpose, we found that samples had to be diluted 1:5 × 10^5^. Camera level was increased until all particles were distinctly visible, not exceeding a particle signal saturation over 20%, and the ideal detection threshold was determined by limiting the blue crossings to 4 per frame. Each sample was measured 5 times with a syringe flow rate of 50 µL/s, at a cell temperature of 25 °C. To minimize data skewing based on single large particles, the number of completed tracks in NTA measurements was always above the minimum value of 1000. Data have been analyzed by the in-built NanoSight Software v3.4.4 and the final histogram of each sample is the result of an average of the multigraph of the five analyzes performed.

### 2.6. HPLC Assessment

Chromatographic experiments were performed by RP-HPLC analysis, with Hewlett-Packard HP1100 system (Palo Alto, CA, USA) consisting of a quaternary pump, continuous vacuum degasser, equipped with a Rheodyne 7125 manual sample injector and a Hewlett-Packard HP UV–vis diode array detector (DAD). A HP ChemStation data system was used for data acquisition and handling. Chromatographic separations were achieved using a LiChroCART Purospher Star RP18-e column (250 mm × 4.6 mm i.d.) (5 µm) (Merck, Darmstad, Germany) combined with a Merck LiChroCART 4-4 LiChrospher 100 RP18 (5 µm) guard column. Elution was carried out isocratically with a mobile phase acetonitrile:water (90:10, *v*/*v*) containing 0.1% trifluoroacetic acid, at a flow rate 2 mL/min, and an injection volume of 20 µL. Detection and chromatographic acquisitions were performed at 406 nm, and peak spectra were recorded by setting the diode array detector from 190 to 500 nm. Identification was carried out by comparing retention times (Rt) and UV absorbance spectra with the reference standard.

### 2.7. Cytotoxicity Studies

Human Embryonic Kidney (HEK) 293 were maintained in complete medium (Dulbecco’s modified Eagle medium; Sigma, Livonia, MI, USA) containing 10% *v*/*v* heat-inactivated fetal bovine serum (Gibco-Invitrogen S.r.l., Carlsbad, CA, USA) and 50 IU/mL penicillin G, 50 μg/mL streptomycin sulphate, and 2 mM L-glutamine (all reagents from Euroclone S.p.A., Milan, Italy). In order to assay changes in cell viability after exposure to liposomal AmB formulations, HEK-293 cells were seeded in quadruplicate in a 96 w plate, 10,000 cells per well in 200 μL of complete medium. After 24 h, the medium was removed, and the cells were exposed for 24 h to: (i) fresh complete medium, (ii) different final drug concentrations (20, 50, 100, 150 μM) from three different stock liposomal formulations of AmB at 4.0 mg/mL (4.32 mM), 2.0 mg/mL (2.16 mM), or 0.2 mg/mL (0.22 mM), either prepared at the time (0 days) or prepared and used after 4 days and stored at two different temperatures, at 4 °C or room temperature (R.T., 25 °C), and (iii) only water for injection or 5% glucose corresponding to the volume collected from the three stock liposomal formulations (4.32, 2.16, 0.22 mM) to test the final drug concentrations of 20, 50, 100, 150, and 200 μM. The viability of HEK-293 cells was evaluated by CyQUANT^®^ Direct Cell Proliferation Assay (Thermo Fisher Scientific, Life Technologies, Milan, Italy) in according to the manufacturer’s instructions. Briefly, after 24 h of treatment, an equal volume of detection reagent was added to the cells in culture and incubated for 1 h at 37 °C. The fluorescence of the samples was measured using the monochromator-based M200 plate reader (Tecan, Männedorf, Switzerland) set at 480/535 nm.

### 2.8. AmBisome^®^ Therapy Optimization: Cost-Saving Analysis

Health expenses, including costs directly related to treatments, are increasing in many countries due to the general progress in medicine that has led to an increase in the average age of populations. The optimization of medication distribution represents an opportunity to improve safety and reduce costs. Indeed, maximizing rational drug use is an important part of the drug control system. At the G. Gaslini children hospital (Genoa, Italy), until May 2022, AmBisome^®^ was given through the nursing station and the pharmacy only supplied the drug to departments. In an attempt to minimize cost, medication floor stocks were removed, and individual prescriptions were managed only by the pharmacists. In order to quantify the amount of pharmaceutical expenditure, an analysis of the direct medical costs of administering AmBisome^®^ was carried out before and after the establishment of the pharmacy-provided medication therapy management.

### 2.9. Statistical Analysis

All the experiments were performed at least three times. Concerning biological studies, each set of experimental conditions for assays was tested in 96-well plates and carried out in quadruplicate. Statistical analyses were determined by *t* test with Welch’s correction two-tailed, or by two-way ANOVA with Bonferroni post hoc test, using GraphPad Prism 5 (GraphPad Software v5.0, San Diego, CA, USA). Differences were significant when *p*-value ranges were: * = *p* < 0.05, ** = *p* < 0.01, *** = *p* < 0.001.

## 3. Results and Discussion

### 3.1. UV–Visible Spectrophotometry

**Figure 3:**
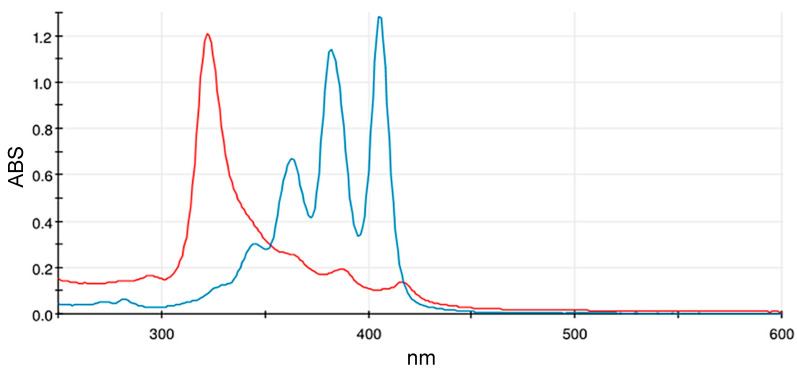
Representative UV–visible spectra of AmBisome^®^ reconstituted in sterile water (0.188 mg/mL, red line) or methanol (0.094 mg/mL, blue line).

AmB possesses a very limited solubility in water at pH 7.4 (below 0.001 mg/mL), although at basic and acidic pH, its solubility is much higher (0.1 mg/mL). This is due to the ionization of the primary amine and carboxylic acid, but the consequent increase in drug degradation recommends its storage as a solid dosage form. For this poor water solubility, AmB has a high tendency to self-assemble in aqueous media by interacting with neighboring polyene chains. In this context, the monitoring and detection of an aggregation state is essential, because it is related to drug activity and toxicity [22]. As highlighted in previous studies, AmB monomers being highly unstable in aqueous medium, tend to gather quickly; it is supposed that the toxic effects (correlated to the increase in membrane permeabilization) could be affected by the presence of aggregates in solution [4], easily detectable by UV–visible spectrophotometric analysis. In this study, the monomeric state was identified by dissolving AmBisome^®^ in methanol at an AmB concentration of 0.094 mg/mL (Figure 3 blue line), with a typical AmB UV–Vis spectrum, characterized by three main peaks at around 363, 383, and 407 nm. The methanolic spectrum mostly reflects the monomeric form of the drug, displaying two main peaks and a third peak of half intensity. Weak absorption peaks can also be observed below 350 nm. As aggregation takes place, absorption bands become prominent between 320 and 350 nm. Indeed, the spectrum of AmBisome^®^ dissolved in water at 0.188 mg/mL showed a characteristic broad peak at 322 nm (Figure 3 red line), ascribable to the hetero-aggregates and three small peaks, typical of the monomeric state.

**Figure 4:**
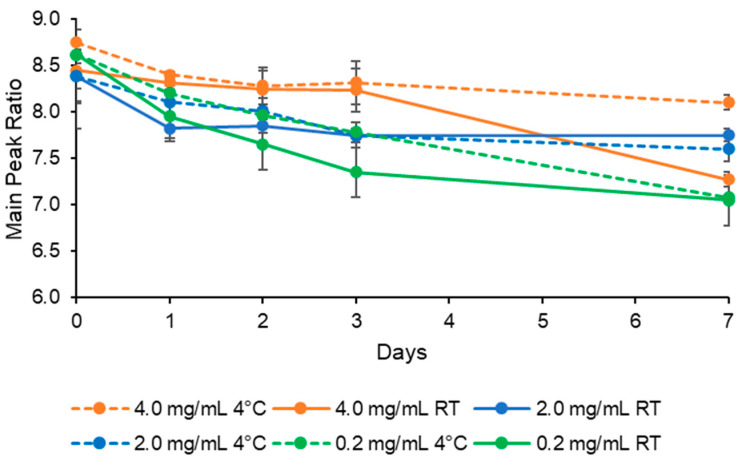
Changes in peak ratio over time under storage conditions. The ratio was calculated by dividing the absorbance of the first peak at 322 nm, characteristic of the oligomer, by the absorbance of the fourth peak at 416 nm, representative of the monomer. The results are reported as the mean ± SD (*n* = 3).

The ratio between the absorption intensity of the first peak (at 322 nm) and the fourth one (at 416 nm) can be used to monitor the aggregation state of AmB. In addition, monitoring the absorption spectra over time could be useful to reveal a drug release, as it can cause a modification of the drug aggregation forms in solution. Therefore, in order to establish if accidental leakage occurred after opening and dilution of AmBisome^®^, in this study, we checked the absorption ratio changing between peak 1 and peak 4. The spectral study was carried out for a week at storage conditions, in terms of temperatures, concentrations and media employed in order to monitor the aggregation state, and thus the physical stability of the formulations. According to the absorption intensity of the peaks, this ratio would be lower for a shift towards monomeric AmB forms and higher for a shift towards oligomeric AmB. Up to one week, all the liposomal formulations containing 4, 2, or 0.2 mg/mL AmB, respectively, kept at RT or 4 °C, displayed no variations in the main peak position, at λmax = 322 ± 1 nm, and the average peak width value was 10 ± 1, suggesting no differences in heterogeneity of species among all the tested formulations [9]. On the contrary, the peak intensity underwent a slight modification, as shown in Figure 4.

The freshly prepared liposomal formulations showed ratios ranging from 8.4 to 8.7, therefore nearly equivalent among each other. However, by increasing storage time after opening, the absorbance ratio between the first peak at 322 nm and the fourth peak at 416 nm decreases over time ranging from 8.1 to 7.1, reflecting some changes in the AmB aggregation state. The increment of the monomeric state occurred gradually and early after 24 h, more evident for the formulations kept at RT than those at 4 °C. Notably, the liposomal formulation at 0.2 mg/mL showed the highest ratio decrement, suggesting the greatest alteration in comparison to the other ones. To date, there are not comparable data in the literature, as a similar study has not been performed yet. Svirkin et al. observed a decrease in peak intensity at 415 nm after heating up to 70 °C, concluding that curing led to tighter aggregation and a decrease in drug release [23], while Liu et al. compared AmBisome^®^ to some generical AmB formulations licensed in India and confirmed the influence of the manufacturing process on the aggregation state, drug release and toxicity [20]. However, they concluded that differences in manufacturing can induce alterations in the supramolecular structure and integrity of the liposomes, which in turn can increase the existence of free AmB, thus explaining the higher toxicity of equivalent formulations. Here, we observed a shift towards monomeric aggregates depending both on storage temperature and initial drug concentration. These changes could be related to a partial drug leakage, as corroborated by the studies of Svirkin and Liu. Indeed, in vitro release studies in clinically relevant media of AmB from AmBisome^®^ revealed a fast drug release, characterized by a first-order kinetic model [24]. On the basis of these considerations, we hypothesized that the shift towards monomer formations followed the release of the drug from the liposome bilayer. Moreover, our samples showed neither any precipitates nor aggregation of liposomes during the period considered, as revealed by the size distribution analysis, suggesting that the arisen monomeric forms could subsequently generate the formation of new and harmful dimers in solution.

### 3.2. In Vitro Colloidal Stability of Nanoformulations

**Figure 5:**
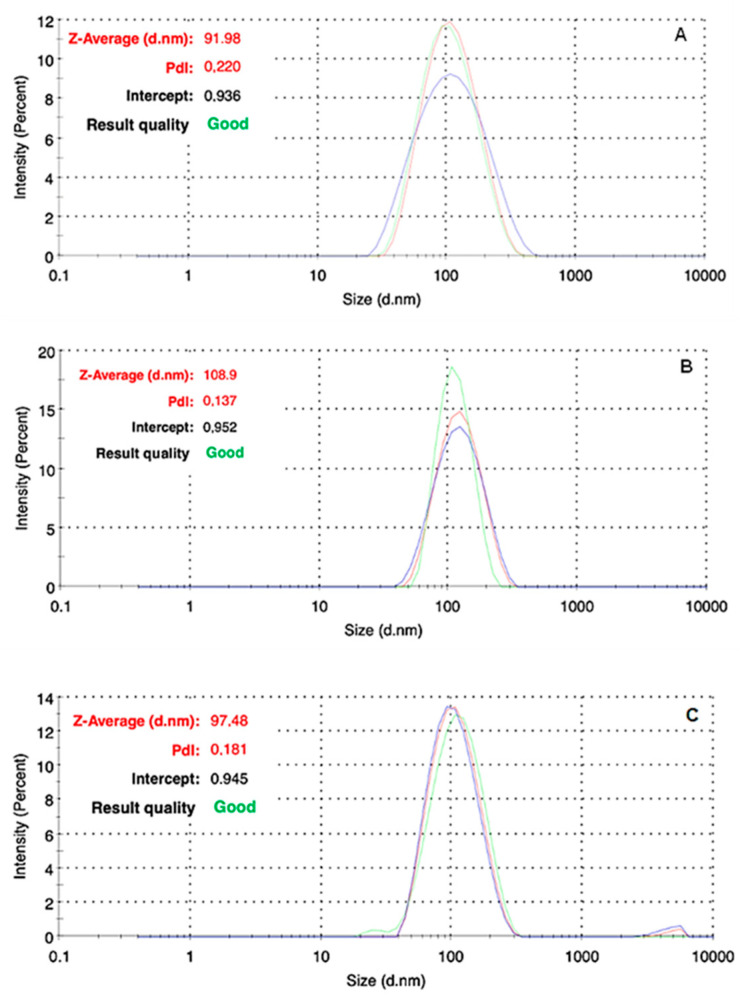
Representative size distributions obtained by DLS of freshly prepared liposomal formulations: (**A**) 4.0 mg/mL AmB in sterile water, (**B**) 0.2 mg/mL, and (**C**) 2.0 mg/mL AmB in 5% glucose solution.

The colloidal stability of reconstituted liposomal suspensions is a prerequisite detrimental for the clinical use of AmBisome^®^. In this study, we assayed the size distribution, PDI, and zeta potential of nanoparticles dispersed in sterile water at 4.0 mg/mL AmB and then diluted with 5% glucose solution, reaching drug concentrations of 2.0 mg/mL and 0.2 mg/mL, according to the manufacturer’s instructions. The study was performed on the three formulations at different time points: immediately after reconstitution or dilution, after 1, 2, and 4 days or 7 days under storage condition (4 °C). Notably, the solution at 4 mg/mL was kept in the fridge for up to 7 days, since the manufacturer granted for its stability for this maximum storage duration. It is important to note that the manufacturer recommends suspension filtration with a 5 µm filter, before dilution with a 5% glucose solution. The mean diameter of the freshly prepared formulations containing 0.2 mg/mL, 2.0 mg/mL, and 4.0 mg/mL AmB were 108.9 nm, 97.5 nm, and 91.9 nm for the samples, respectively (Figure 5). All liposomal formulations have mainly similar sizes around 100 nm with a polydispersity index of around 0.2.

These data are quite in agreement with those provided by Ye et al., whose findings showed an average diameter of 131 nm for a 1.0 mg/mL AmB diluted with a 5% dextrose solution [25], and more in line with the results exposed by Liu et al., which provided a value of 103 nm for the 4.0 mg/mL formulation [20]. As mentioned above, we monitored the properties of the liposomal formulations by DLS at specific time points to investigate changes occurring on the temporal scale. As reported in Table 2, the DLS technique did not evidence any significant variation within each type of sample stored at 4 °C up to 4 days or 1 week, thus confirming the high stability of the lipid bilayer of the delivery system. We furtherly prolonged the time of observation up to 14 days, but no significant changes were detected.

When analyzed by NTA, the same formulations always had a mean diameter slightly smaller than that determined by DLS (Table 2, Figure S1). This result is in contrast with the findings of Liu et al. [20], whose study revealed always larger values for NTA-based measurements. This slight discrepancy among the two research studies may lie in the difficulty in characterizing nano-systems like liposomes linked to the complexity of manufacturing processes and reproducibility of the scale-up, all factors that may influence the uniformity of the batches. Moreover, while the sizes remained similar over time for the two formulations diluted in 5% glucose, the liposomal AmB 4 mg/mL suspension showed an increase in the diameter from 87 nm to 104 nm. This increase in diameter could be related to the external osmotic pressure to which the liposomes are subjected, being dispersed in a hypotonic medium (sterile water) [26]. Regarding the superficial electric charge measured by DLS, we found that the zeta potential hardly changed with time within 4 days or 1 week (Table 2, Figure S2). AmBisome^®^ consists of three lipids: hydrogenated soybean phosphatidylcholine (HSPC), distearoyl phosphatidylglycerol (DSPG), and cholesterol. DSPG is an anionic phospholipid, while HSPC and AmB are zwitterionic molecules; therefore, the surface of the liposomes became negatively charged, like most liposomal products on the market. This negative net charge assures colloidal stability and avoids flocculation processes. In fact, during the time period considered, we observed neither formation of precipitates due to drug desolvation, nor clusters of nanoparticles as confirmed by NTA. In this study, the number of particles in suspension remained almost constant or at least of the same magnitude order for each sample, highlighting the high stability of the supramolecular structure of the liposomes.

### 3.3. HPLC Analysis

It has been reported that AmB suffers from degradation when exposed to light, heat, radical initiators, and extreme pH conditions. The by-products have not been isolated yet, but the most probable reactions involve the highly conjugated polyene backbone, similar to a lipid oxidation [27]. Moreover, previous studies suggested that the degradation products may increase drug toxicity, such as acute hemolysis [28]. The chromatographic conditions used in this study were based on those reported by Espada et al., with some changes [29]. The detection wavelength for AmB and its related impurities, eventually present during in-use conditions storage, was set at 406 nm, as already proposed for the detection of some degradation products [27]. Linearity was maintained with stock solutions in methanol ranging from 38 to 360 µg/mL AmB. The calibration curve was generated from the average peak areas from three independent experiments, and the regression equation of the line was (Y = 32985X + 120.07) with a correlation coefficient of 0.9999 (Figure S3). With the chromatographic conditions adopted, the main peak of AmB appeared around 7.5632 ± 0.1782 min (Figure S4).

**Figure 6:**
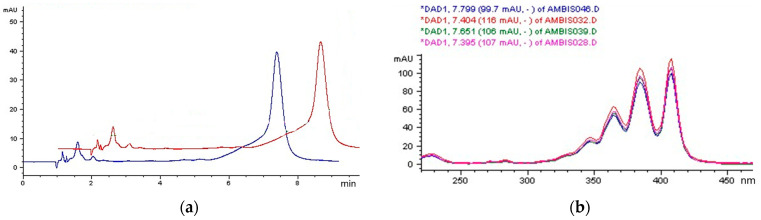
(**a**) Representative HPLC chromatograms of freshly prepared 4.0 mg/mL AmB in sterile water (blue line) and after 7 days (red line). (**b**) Overlaid UV–Vis spectra of AmB peak (Rt = 7.5632 min) obtained by DAD related to freshly prepared 4.0 mg/mL AmB suspension in sterile water (blue line), 0.2 mg/mL AmB in 5% glucose solution (pink line) and after 7 days at 4 °C (red line and green lines, respectively).

In order to determine if the different AmB formulations containing 4.0, 2.0, or 0.2 mg/mL AmB underwent some degradative reactions under storage conditions, HPLC-DAD analyses were performed on AmB samples after 1, 2, 3, and 7 days from the reconstitution of the lyophilized sample. Since at hospital the AmBisome^®^ formulations, managed by the pharmacists for dose adjustments and administration to different patients, were stored in a fridge, we selected 4 °C for our stability studies. The study of recorded chromatograms, peak identity, and peak purity up to 7 days did not show any remarkable variation from the freshly prepared formulations (Figure 6), thus confirming that just storing the drug at 4 °C prevents chemical reactions and by-product formation. However, during the time period considered, the chemical stability shown by the drug in these conditions did not correspond to an unmodified aggregation state, as revealed by spectrophotometric analysis. This behavior suggests that, when the drug is kept in solution, its rearrangement (intercalated or outside the phospholipid by-layer) does not affect any chemical modifications of the molecule.

### 3.4. In Vitro Toxicity Evaluation of AmB Formulations

**Figure 7:**
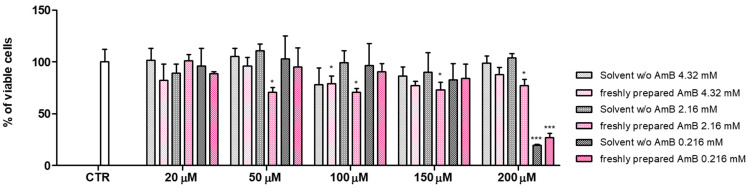
Three different stock concentrations of formulations containing AmB 4.32 mM, 2.16 mM, or 0.22 mM were prepared and used at different final drug concentrations 20, 50, 100, and 150 µM to treat HEK-293 cells for 24 h. Equivalent amounts of solvent without drug were used as blanks for each sample tested. Data were acquired by the use of the TECAN microplate reader, Infinite 200 (Tecan Life Sciences) set (i.e., 480/535 nm). Data are expressed as the mean ± SD of three independent experiments (* *p* < 0.05, *** *p* < 0.001 vs. CTR). CTR—control; µM—micromolar; mM—millimolar; h—hours.

The in vitro toxicity of the three freshly prepared liposomal formulations, containing AmB 4.0 mg/mL (4.32 mM) in sterile water, or AmB 2.0 mg/mL (2.16 mM) in 5% glucose solution, or AmB 0.2 mg/mL (0.22 mM) in 5% glucose solution, were evaluated in HEK-293 cells due to the high incidence of nephrotoxicity exerted by AmB. We performed for the first time a time- and dose-dependent study on cell viability of the liposomal AmB formulations as they are prepared in the hospital pharmacy and then administered to patients, aiming to investigate if the handling and the storage time could exert some unexpected drug effects. Firstly, the cells were exposed for 24 h to freshly prepared formulations with or without the drug to assess if the variation in the concentration of the medium constituents could be responsible itself for some toxic effects or for a decrease in cell proliferation. Therefore, the cells were exposed to the same volume of buffer (sterile water or 5% glucose) present in the loaded formulations to evaluate possible alterations in cell viability linked to osmotic effects or lack of nutrients. As shown in Figure 7, the presence of the buffer reduces in a significative manner the cell vitality only in the case of 0.22 mM AmB formulation for a drug concentration of 200 µM. In this case, to reach that concentration of drug, the volume of the buffer added was at its highest level in comparison with the other samples. On the contrary, at all the other concentrations tested and for the other two formulations, the effects on survival rate are ascribable always to the presence of the drug. Notably, among the liposomal AmB formulations, the AmB 2.16 mM began to be toxic at 50 µM, while maintaining the same level of toxicity also at the subsequent concentrations tested, suggesting a non-dose-dependent mechanism. The formulation of AmB 4.32 mM in sterile water seems less toxic, as it exerted a slight influence on cell vitality only at 100 µM. On the contrary, the less concentrated AmB liposomal formulation (0.22 mM) showed a remarkable toxicity at the maximum concentration tested, but this effect is attributable to the excess of solvent added. Therefore, these results highlighted some differences among the formulations regarding their biological activity. It is noteworthy that the encapsulation of the drug into the liposome bilayer significantly reduces AmB cytotoxicity; indeed, the free drug is highly cytotoxic (>80% cell death) at a concentration of about 2 µM [30]. However, our study demonstrated that the liposomal formulations are not equivalent from the toxicological point of view. In fact, at the same drug concentration, the effects of the three liposomal formulations are different. If we exclude the highest drug concentration (200 µM) at which the effect of the excessive presence of solvent is not negligible, for all other concentrations the toxicity of the liposomal formulation follows the order AmB 4.32 mM < AmB 0.22 mM < AmB 2.16 mM. These results give a new insight into the safety of using nanosized drug-delivery systems, confirming the difficulties of standardization of liposomes [31].

**Figure 8:**
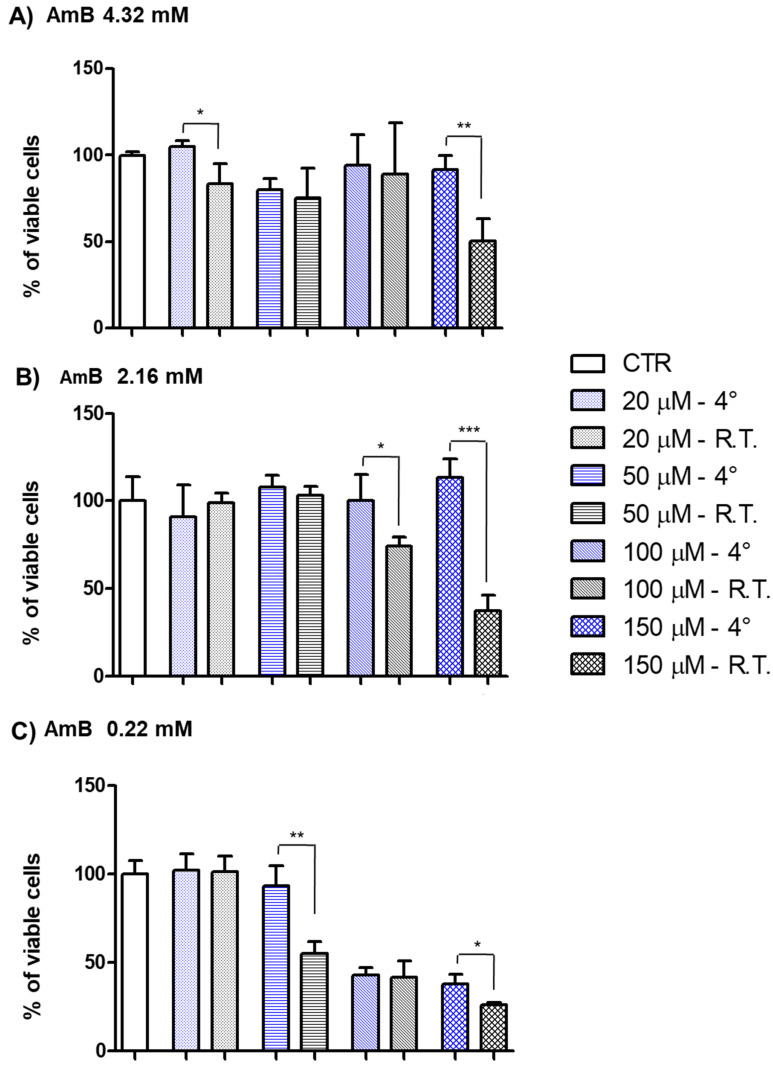
Three different stock concentrations of liposomal formulations containing (**A**) 4.32 mM, (**B**) 2.16 mM, or (**C**) 0.22 mM AmB were prepared and stored for 4 days at 4° (blue bars) or RT (black bars), and used at different final drug concentrations of 20, 50, 100, and 150 µM to treat HEK-293 cells for 24 h. Data were acquired using the TECAN microplate reader, Infinite 200 (Tecan Life Sciences) set (i.e., 480/535 nm). Data are expressed as the mean ± SD of three independent experiments (* *p* < 0.05, ** *p* < 0.01, *** *p* < 0.001, of 4 °C vs. RT for each final concentration). CTR—control; µM—micromolar; mM—millimolar; h—hours.

In order to verify if storage conditions could further alter the biological activity of AmBisome^®^, we treated the cells for 24 h with the three liposomal formulations maintained for 4 days in the fridge or at RT On the basis of the results of the previous experiment, we selected only the concentrations whose blanks could not interfere with the results, therefore the 200 µM drug concentration was excluded. As shown in Figure 8, the cytotoxicity of the formulations was influenced by the storage temperature, and generally, the suspensions stored at RT were always more cytotoxic in comparison with those maintained in the fridge. Notably, the discrepancy between the storage at RT and at 4 °C was significantly more remarkable at the highest concentration (150 µM) for the formulations of AmB 4.32 mM in sterile water and for AmB 2.16 mM in 5% glucose. Probably, at the highest concentrations, the amount of free drug present in solution increases, reaching the capability of forming dimers. Indeed, when only monomers are present in solution, the drug affects only ergosterol-rich membranes, and it is less toxic. Conversely, the onset of dimerization, which is estimated to occur early at about 0.41 µM, reduces drug selectivity and increases drug toxicity towards mammalian cells [32]. Therefore, we can deduce that, at R.T., a more markedly drug leakage occurs; consequently, it is recommended not to leave the formulations outside the fridge. Curiously, the less concentrated formulation (AmB 0.22 mM) had a different behavior. In this case, the onset of a significant toxicity started at 50 µM for the sample maintained at RT and at 100 µM for the sample maintained at 4 °C. These data highlight the greater stability of the supramolecular system when stored at low temperatures, as revealed also by spectrophotometric analysis, but also show a remarkable difference among the three formulations. Particularly, it was evident that the 0.22 mM formulation after 4 days of storage was more effective in reducing cell vitality. Since the AmB toxicity is always related to its rearrangement in solution, the presence of free form of the drug outside the liposome’s bilayer may be the main cause of the detrimental effect exerted on the cell viability by the drug. Our data indicate that the most diluted suspension was also the most toxic, probably due to its greater colloidal instability. These results are consistent with those obtained from the spectrophotometric analysis, which revealed that the AmBisome^®^ dilution to the lowest concentration employed in the clinical administration was characterized by the presence of greater changes in the aggregation state. The influence of dilution on the colloidal stability of a supramolecular system is well recognized [33]. In addition, the greater instability of the most diluted formulation is consistent with the indications of storage reported in the SmPC, where it is stated that the stability at 4 °C is granted up to 7 days for AmB 4.32 mM and 2.16 mM, and only up to 4 days for AmB 0.22 mM formulation (Table 1). We confirmed that the dilution by ten times can transform the AmBisome^®^ suspension into a less safe formulation. Consequently, pharmacists have to pay more attention to the clinical administrations involving the youngest patients, who need low dosages. In summary, it would be better to consume the more diluted formulations in a shorter period of time, considering that they are administered to the more vulnerable patients.

### 3.5. The Impact of the Centralized Compounding of AmBisome^®^ at the G. Gaslini Children Hospital Pharmacy

The shifts towards personalized medicines on the one hand and the pressure on national health budgets and the shortage of medicines on the other have increased more and more, compounding the activity of the hospital pharmacy [34]. Among the procedures carried out by the pharmacists, the reconstitution of intravenous medicines, such as anti-infectives, analgesics, and anti-emetics represent one of the most frequent activities. Moreover, the European Association of Hospital Pharmacists (EAHP) has greatly encouraged the involvement of hospital pharmacies in reconstitution practices and in the creation of a centralized reconstitution unit, according to the Council of Europe resolution on good reconstitution practices in healthcare establishments for medicinal products for parenteral use [35]. The tasks of the hospital pharmacist in this regard include risk management, supervision of the work quality, and overall work planning.

AmBisome^®^’s relatively frequent use is related to its broad spectrum of activity and the limited number of alternatives, as only a few antifungal agents are licensed for use in children. Published multicenter experiences have demonstrated that liposomal AmB is the preferred antifungal choice for probable and proven invasive fungal disease in pediatric patients [36]. The safety and efficacy of AmBisome^®^ has been established in pediatric patients aged between one month to 18 years old. Dosages can be calculated on the same per-kg body weight basis as for adults, and the maximum tolerated dose is 10 mg/kg/day, but with an increasing occurrence of hypokalemia and infusion-related vomiting with doses above 5 mg/kg/day [37].

For better monitoring of the routine drug prescriptions and to face the necessity of addressing the medication to the patient needs, the pharmacy of the G. Gaslini children hospital has opted for the centralized preparation of AmBisome^®^ since May 2022. It has been demonstrated that the supervision of hospital pharmacists in the preparation of ready-to-administer products plays an important role in improving quality of care, patient safety, and cost saving [38]. Prior to May 2022, the reconstitution of the drug could take place on the ward. Our aim was to evaluate if the strategy of centralization would lead to a cost-effective drug, by means of waste-reduction, without any detrimental effects on patient care.

The expenditure associated with AmBisome^®^ utilization is a direct and variable cost, depending on patient volume. For the quantification of the direct cost associated with AmBisome^®^, data were gathered before and after the centralization of the drug preparation. Firstly, we collected data regarding the number of vials used for all ongoing and set up therapies (Table 3). Then, we calculated the mean number of vials of AmBisome^®^ employed per therapy, dividing the number of vials used by the number of treatments provided monthly. Then, the cost per treatment was obtained by multiplying the number of vials per treatment by the cost of 1 unit of AmBisome^®^ at the price purchased by the hospital pharmacy (EUR 146.80). As shown in Table 3, when the medication was distributed by a ward stock system, the cost per treatment was EUR 814.74; meanwhile, when the reconstitution of the drug was carried out only at the pharmacy, the price remarkably decreased to EUR 336.17, thus leading to a cost saving of EUR 478.57 per therapy.

## 4. Conclusions

In this study, we investigated the stability under storage conditions of three different AmBisome^®^ formulations, corresponding to the dilutions used by the hospital staff for clinical administration: AmB 4 mg/mL (4.32 mM) in sterile water, AmB 2 mg/mL (2.16 mM), and AmB 0.2 mg/mL (0.22 mM) in 5% glucose solution. The formulations were stored at RT or at 4 °C up to 1 week and analyzed for their colloidal and chemical stabilities. The colloidal stability of the three formulations revealed a reposition of the drug during storage, starting early after 24 h and being particularly evident at RT. Indeed, we observed an increase in the monomeric drug forms, probably originating from the onset of free drug molecules released from the liposomal delivery system. The monomeric forms, having a low water solubility at submicromolar concentrations, quickly shift towards the more toxic dimeric forms. The modifications of the aggregation state were more evident for the 0.22 µM AmB formulation, highlighting a greater colloidal instability of the most diluted formulation. The biological results were in line with the changes revealed by spectrophotometric analysis, since the 0.22 µM AmB formulation was the most effective in reducing cell viability. This increased detrimental effect on cells was not ascribable to the onset of drug byproducts, as confirmed by HPLC analysis, but it probably depends on the growing presence of free drug forms of the drug in solution. Thus, our results demonstrated the difficulties related to handling nanometric supramolecular systems, since from the same batch of AmBisome^®^, we obtained diverse-diluted liposomal formulations highly and differently influenced by drug concentration, time, and temperature of storage. Only the colloidal stability of the liposomal suspensions remained almost constant for up to 7 days at 4 °C.

Therefore, the compounding pharmacist has to pay much attention when handling this medication. On the basis of our results, the pharmacist should avoid RT storage and consider that more diluted samples are prone to physical instability and greater toxicity. This aspect is of particular relevance, especially in a pediatric hospital, younger patients being those requiring the lowest dosages. So, in light of these considerations, the centralization of AmBisome^®^ dilution at the pharmacy department is absolutely essential for assuring patient safety, medication quality, and cost saving, as we have demonstrated by the analysis of drug expense per therapy carried out at the G. Gaslini children hospital.

## Figures and Tables

**Table 1:** In-use storage times after opening AmBisome^®^ suggested by the manufacturer and employed in the hospital pharmacy.

Diluent	Concentration of AmB (mg/mL)	Maximum Duration of Storage at 4 °C		Maximum Duration of Storage at 25 °C	
		Manufacturer	Hospital	Manufacturer	Hospital
Water for injection	4.0	7 days	7 days	24 h	N ^1^
5% glucose	2.0	7 days	24 h	48 h	N
5% glucose	0.2	4 days	4 days	24 h	N

^1^ N = not in-use condition.

**Table 2:** Size distribution and zeta potential results of different liposomal AmB formulations detected by the DLS (dynamic light scattering) and NTA (nanoparticle tracking analysis) systems. Data represented as mean ± SD (*n* = 3).

Sample	NTA System				Dynamic Light Scattering			Storage at 4 °C
	Concentration (Particles/mL)	Mean Diameter (nm)	Mode (nm)	Percentile Values	Z-Average (nm)	PDI	Zeta Potential (mV)	Time (Days)
Liposomal AmB 4 mg/mL in sterile water	3.94 × 10^13^ ± 1.43 × 10^12^	87.0 ± 1.0	68.3 ± 0.7	D10 = 58.8 ± 0.2	91.9 ± 7.2	0.220	−32.6 ± 7.3	0
				D50 = 81.4 ± 1.1				
				D90 = 122.4 ± 0.8				
	1.33 × 10^14^ ± 6.24 × 10^12^	92.6 ± 1.1 *	75.8 ± 2.0	D10 = 64.2 ± 0.5	93.9 ± 5.2	0.189	−33.9 ± 6.7	2
				D50 = 84.0 ± 1.1				
				D90 = 135.2 ± 2.0				
	1.28 × 10^14^ ± 6.81 × 10^12^	104.0 ± 3.5 **	81.5 ± 2.5	D10 = 62.2 ± 2.2	92.9 ± 4.7	0.210	−32.4 ± 7.2	7
				D50 = 96.9 ± 3.3				
				D90 = 154.1 ± 3.7				
Liposomal AmB 2 mg/mL in 5% glucose	8.47 × 10^13^ ± 1.80 × 10^12^	89.7 ± 1.4	75.4 ± 0.8	D10 = 63.6 ± 1.9	97.5 ± 8.4	0.181	−41.4 ± 10.9	0
				D50 = 82.5 ± 0.8				
				D90 = 128.7 ± 1.7				
	7.06 × 10^13^ ± 1.43 × 10^12^	91.9 ± 0.3	78.9 ± 3.2	D10 = 64.0 ± 0.9	102.2 ± 4.6	0.150	−42.0 ± 11.5	1
				D50 = 87.3 ± 0.5				
				D90 = 125.3 ± 0.8				
	8.18 × 10^12^ ± 2.11 × 10^11^	90.9 ± 1.2	79.3 ± 0.8	D10 = 66.2 ± 2.1	99.3 ± 5.2	0.146	−42.2 ± 10.9	2
				D50 = 84.6 ± 0.7				
				D90 = 127.1 ± 3.2				
	8.80 × 10^13^ ± 1.68 × 10^12^	85.1 ± 0.6 *	73.6 ± 0.3	D10 = 62.2 ± 0.6	99.3 ± 6.5	0.160	−40.9 ± 10.7	4
				D50 = 79.5 ± 0.6				
				D90 = 117.0 ± 2.6				
Liposomal AmB 0.2 mg/mL in 5% glucose	9.48 × 10^12^ ± 3.17 × 10^11^	91.1 ± 1.1	78.8 ± 2.5	D10 = 63.5 ± 1.1	108.9 ± 7.1	0.137	−44.4 ± 15.9	0
				D50 = 87.0 ± 0.8				
				D90 = 130.0 ± 2.6				
	9.19 × 10^12^ ± 1.92 × 10^11^	87.6 ± 0.3	77.3 ± 2.1	D10 = 63.9 ± 0.4	112.4 ± 5.6	0.136	−45.9 ± 14.5	1
				D50 = 82.7 ± 0.6				
				D90 = 118.1 ± 1.9				
	8.20 × 10^12^ ± 6.82 × 10^10^	90.7 ± 0.5	77.0 ± 1.8	D10 = 64.9 ± 0.9	109.2 ± 8.2	0.133	−46.5 ± 13.9	2
				D50 = 83.2 ± 0.5				
				D90 = 131.2 ± 1.2				
	7.47 × 10^12^ ± 2.62 × 10^11^	88.6 ± 0.6	75.9 ± 1.4	D10 = 64.5 ± 0.7	109.0 ± 6.5	0.131	−45.7 ± 16.0	4
				D50 = 82.8 ± 0.9				
				D90 = 124.0 ± 2.7				

* *p* < 0.05, ** *p* < 0.01.

**Table 3:** Analysis of the cost per therapy associated with the administration of AmBisome^®^ at the G. Gaslini children hospital before and after the decision of centralization of drug reconstitution.

Time Period		Vials of AmBisome^®^ Used	Number of Therapies	Vials per Therapy	Cost per Therapy (€)
Before	January 2022	418	72	5.55 ± 0.35	814.74
	February 2022	306	58		
After	June 2022	152	83	2.29 ± 0.38	336.17
	July 2022	201	91		
	August 2022	251	99		
	September 2022	280	106		

## Data Availability

Data are contained within the article and Supplementary Materials.

## Supplementary Materials

The following supporting information can be downloaded at: https://www.mdpi.com/article/10.3390/pharmaceutics16040466/s1: Figure S1—representative size distribution obtained by NTA system of freshly prepared liposomal formulations; Figure S2—representative distribution of Zeta potentials of freshly prepared liposomal formulations; Figure S3—calibration curve of the HPLC assay method; Figure S4—chromatograms of the AmB calibration standard methanol solutions.
