# Supplementary material for: AmBisome® Formulations for Pediatrics: Stability, Cytotoxicity, and Cost-Effectiveness Studies
Source: Pharmaceutics. 2024 Mar 27;16(4):466. doi: 10.3390/pharmaceutics16040466 (PMC11054559; doi:10.3390/pharmaceutics16040466)
Supplement: Supplementary file 1 [file pharmaceutics-16-00466-s001.zip › pharmaceutics-2909567-supplementary.pdf]

pharmaceutics

MDPI

Supplementary information

AmBisome® Formulations for Pediatrics: Stability, Cytotoxicity, and Cost-Effectiveness Studies

Guendalina Zuccari [1,*], Carla Villa [1], Valentina Iurilli [2], Paola Barabino [2], Alessia Zorzoli [3], Danilo Marimpietri [3], Debora Caviglia [1] and Eleonora Russo [1,*]

[1] Department of Pharmacy, University of Genoa, Viale Benedetto XV, 16132 Genoa, Italy; carla.villa@unige.it (C.V.); debora.caviglia@edu.unige.it (D.C.)
[2] UOC—Unità Operativa Complessa, IRCCS Istituto Giannina Gaslini, via Gerolamo Gaslini 5, 16147 Genoa, Italy; valentinaiurilli@gaslini.org (V.I.); paolabarabino@gaslini.org (P.B.)
[3] Stem Cell Laboratory and Cell Therapy Center, IRCCS Istituto Giannina Gaslini, via Gerolamo Gaslini 5, 16147 Genoa, Italy; alessiazorzoli@gaslini.org (A.Z.); danilomarimpietri@gaslini.org (D.M.)
* Correspondence: guendalina.zuccari@unige.it (G.Z.); eleonora.russo@unige.it (E.R.)

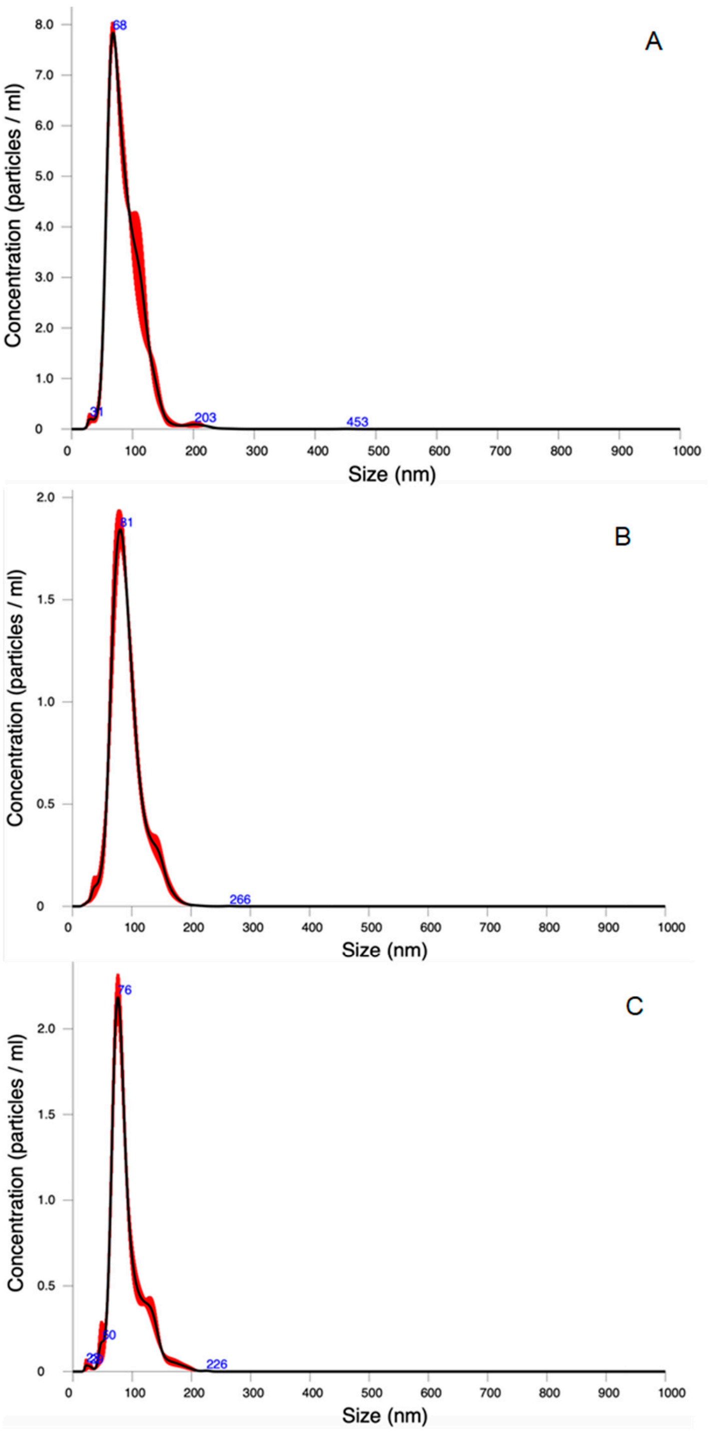

Figure S1. Representative size distribution obtained by NTA system of freshly prepared liposomal formulations: (A) 4.0 mg/mL AmB in sterile water, (B) 0.2 mg/mL and (C) 2.0 mg/mL AmB in 5% glucose solution.

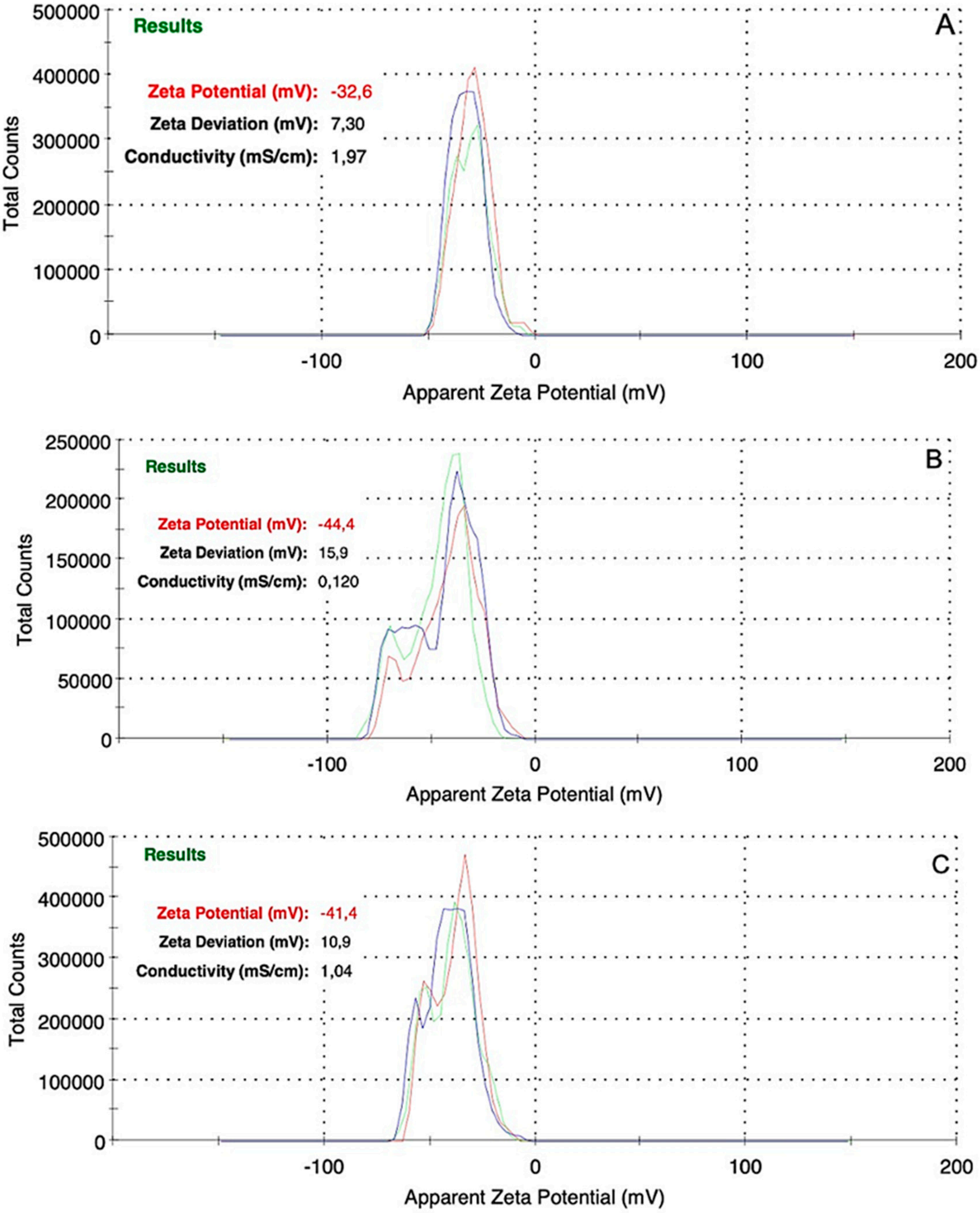

Figure S2. Representative distribution of zeta potentials of freshly prepared liposomal formulations: (A) 4.0 mg/mL AmB in sterile water, (B) 0.2 mg/mL and (C) 2.0 mg/mL AmB in 5% glucose solution.

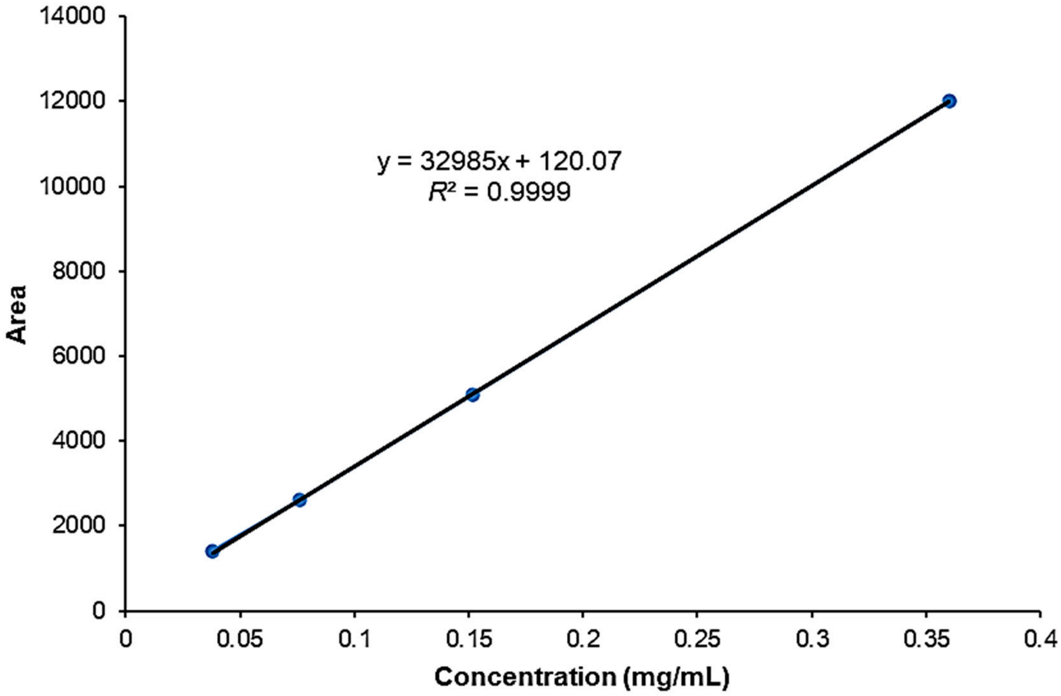

Figure S3. Calibration curve of the HPLC assay method. Calibration standards are 0.038, 0.076, 0.152, 0.36 mg/mL of AmB in methanol solution.

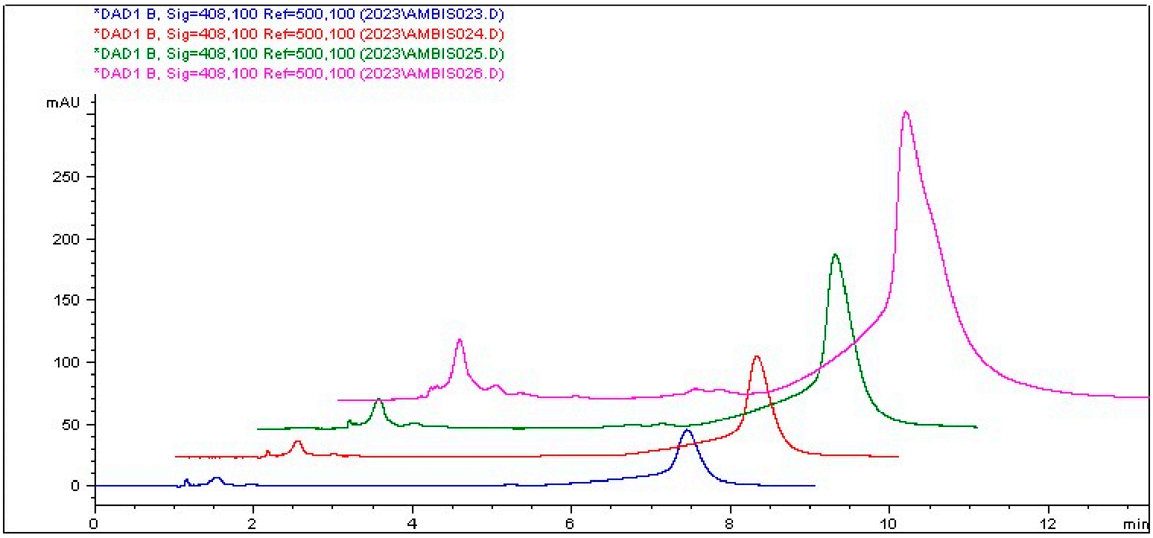

Figure S4. Chromatograms of the AmB calibration standard methanol solutions: 0.038 (blue line), 0.076 (red line), 0.152 (green line), 0.36 (pink line) mg/mL.
